# Editorial: Interactions of nanoparticles with and within living organisms—What can we learn to improve efficacy of nanomedicine?

**DOI:** 10.3389/fmolb.2022.1044063

**Published:** 2022-10-18

**Authors:** N. Helge Meyer, Claudia Corbo, Guillermo R. Castro, Goran Stjepanovic, Giada G. Genchi, Valerio Marino

**Affiliations:** ^1^ Department of Human Medicine, University Hospital of General and Visceral Surgery, University of Oldenburg, Milan, Italy; ^2^ Center for Nanomedicine NANOMIB, Department of Medicine and Surgery, University of Milan Bicocca, Milan, Italy; ^3^ IRCCS Istituto Ortopedico Galeazzi, Milan, Italy; ^4^ Max Planck Laboratory for Structural Biology, Chemistry and Molecular Biophysics of Rosario (MPLbioR,UNR-MPIbpC), Partner Laboratory of the Max Planck Institute for Biophysical Chemistry (MPIbpC,MPG), Center for Interdisciplinary Studies (CEI—CONICET), National University of Rosario, Rosario, Argentina; ^5^ Nanomedicine Nucleus, Center for Natural and Human Sciences, Federal University of ABC, Santo André, Brazil; ^6^ School of Medicine, Kobilka Institute of Innovative Drug Discovery, The Chinese University of Hong Kong, Shenzhen, China; ^7^ Smart Bio-Interfaces, Center for Materials Interfaces, Istituto Italiano di Tecnologia, Pontedera, Italy; ^8^ Section of Biological Chemistry, Department of Neurosciences, Biomedicine and Movement Sciences, University of Verona, Verona, Italy

**Keywords:** nanoparticles, nanomedicine, green synthesis, NMR, nanosuspensions, pancreatic cancer, cancer immunotherapy, targeted delivery

Nanoparticles (NPs) are on the verge of being established as a mainstay of modern medicine. Currently, numerous synthetic nanomaterials derived from various organic or inorganic materials such as lipids, proteins, synthetic/natural polymer, and metals are available for therapeutic and diagnostic purposes. Due to their structural versatility, storage stability, and ease of functionalization, NPs have extensive fields of applications spanning from environmental science to medicine. In medicine, NPs are incessantly being improved for drug delivery, tissue engineering, and diseases diagnosis, just to name a few. However, *in vitro* and animal models of NP applications often fail to translate successfully to clinical settings, highlighting a lack of understanding in the interaction of NPs with living systems. In this Research Topic, the multiple aspects of this dilemma are examined from different angles by researchers across a range of disciplines.

The global outbreak of SARS-CoV2 has held us hostage for the past 2 years. A novel class of NP-based mRNA vaccines has emerged as a highly effective weapon in the fight against the virus. (Schoenmaker et al., 2021). However, the development of antiviral therapies against SARS-CoV2 remains critical. Stanisic et al. present a cost-effective, bio-based synthesis of antiviral silver NPs and 3D flexible nanostructure composite materials, which effectively suppressed infection of fibroblasts with a murine coronavirus as a proxy for SARS-CoV-2. Furthermore, the authors present an innovative approach to tracing coronavirus infection *in vitro* utilizing high-resolution magic-angle spinning NMR spectroscopy.

It is believed that plants were used for therapeutic purposes for at least 60.000 years, based on fossil records (Solecki, 1975). In modern medicine plants still serve as valuable sources in drug development, *e.g*. for the isolation of novel active compounds or the production of therapeutic extracts (Fabricant and Farnsworth, 2001). Medical use of plant extracts, however, is challenging due to complex compositions, risk of toxicity, and instability issues. In this regard, NP formulations have the potential to reduce plant extract toxicity and facilitate targeted delivery. Emanet et al. encapsulated hazelnut extracts, known to have antioxidant properties, in nanostructured lipid carriers which demonstrated optimal cytocompatibility and excellent antioxidant activity in a human dermal fibroblast cell culture model. Ali et al. present a novel strategy to generate and comprehensively characterize nanosuspension from Nigella (N.) sativa, an annual blooming herb rich in thymoquinone (Hannan et al., 2021). Nanosuspension of N. sativa had increased bioavailability of bioactive compounds, consequently showing higher biochemical activity as compared to conventional ethanolic extracts. This study highlights the development of environmentally friendly synthesis of nanosuspensions for studying the enhanced bioactivities.

NPs can specifically be designed to overcome several limitations of free therapeutics. In terms of targeted delivery, they offer immense potential to bypass biological barriers (Mitchell et al., 2021). The oral mucosal barrier–beyond physically limiting molecular uptake–also has a transport and metabolic component, responsible for regulating influx, efflux, enzymatic modification and degradation of substances (Bierbaumer et al., 2018). Jeitler et al. aimed at optimizing the composition of lipid-structured NPs in order to enhance their efficiency safety and uptake by buccal epithelial cells. By careful evaluation of NP-to-cell interactions, they identified caveolin-mediated endocytosis as a major uptake route. These findings might guide prospective biopharmaceutical studies.

Nanomaterials have been used extensively for the development of novel cancer therapeutics (Aghebati-Maleki et al., 2020). Particularly, NPs display enhanced targeting capabilities and prolonged retention in the tumor microenvironment of many cancers in ways that can potentially overcome the limitations of immunotherapy. A review by Noubissi Nzeteu et al. recapitulates in depth NP-based immunotherapy for pancreatic cancer and provides an outlook on future treatment possibilities.

In conclusion, this collection of related articles highlights novel strategies to understand the physiological and pathological properties of NPs. A comprehensive understanding of NP behavior in clinical settings will eventually be obtained from cell-based and *in vivo* studies, accelerating the introduction of nanomedicine into clinical practice.
